# Integration of circulating tumor cell and neutrophil-lymphocyte ratio to identify high-risk metastatic castration-resistant prostate cancer patients

**DOI:** 10.1186/s12885-021-08405-3

**Published:** 2021-06-02

**Authors:** Weelic Chong, Zhenchao Zhang, Rui Luo, Jian Gu, Jianqing Lin, Qiang Wei, Bingshan Li, Ronald Myers, Grace Lu-Yao, William Kevin Kelly, Chun Wang, Hushan Yang

**Affiliations:** 1 Department of Medical Oncology, Sidney Kimmel Cancer Center, Thomas Jefferson University, Philadelphia, PA 19107 USA; 2 Department of Epidemiology, MD Anderson Cancer Center, Houston, TX 77030 USA; 3 Department of Medicine, GW Cancer Center, George Washington University, Washington, DC 20037 USA; 4 Department of Molecular Physiology and Biophysics, Vanderbilt University, Nashville, TN 37235 USA

**Keywords:** Circulating tumor cell, Neutrophil-lymphocyte ratio, Platelet-lymphocyte ratio, Metastatic castration-resistant prostate cancer, Prognosis

## Abstract

**Background:**

The neutrophil-lymphocyte ratio (NLR), platelet-lymphocyte ratio (PLR), and circulating tumor cells (CTCs) have been associated with survival in castration-resistant prostate cancer (CRPC). However, no study has examined the prognostic value of NLR and PLR in the context of CTCs.

**Methods:**

Baseline CTCs from mCRPC patients were enumerated using the CellSearch System. Baseline NLR and PLR values were calculated using the data from routine complete blood counts. The associations of CTC, NLR, and PLR values, individually and jointly, with progression-free survival (PFS) and overall survival (OS), were evaluated using Kaplan-Meier analysis, as well as univariate and multivariate Cox models.

**Results:**

CTCs were detected in 37 (58.7%) of 63 mCRPC patients, and among them, 16 (25.4%) had ≥5 CTCs. The presence of CTCs was significantly associated with a 4.02-fold increased risk for progression and a 3.72-fold increased risk of death during a median follow-up of 17.6 months. OS was shorter among patients with high levels of NLR or PLR than those with low levels (log-rank *P* = 0.023 and 0.077). Neither NLR nor PLR was individually associated with PFS. Among the 37 patients with detectable CTCs, those with a high NLR had significantly shorter OS (log-rank *P* = 0.024); however, among the 26 patients without CTCs, the OS difference between high- and low-NLR groups was not statistically significant. Compared to the patients with CTCs and low NLR, those with CTCs and high levels of NLR had a 3.79-fold risk of death (*P* = 0.036). This association remained significant after adjusting for covariates (*P* = 0.031). Combination analyses of CTC and PLR did not yield significant results.

**Conclusion:**

Among patients with detectable CTCs, the use of NLR could further classify patients into different risk groups, suggesting a complementary role for NLR in CTC-based prognostic stratification in mCRPC.

**Supplementary Information:**

The online version contains supplementary material available at 10.1186/s12885-021-08405-3.

## Introduction

Androgen deprivation therapy (ADT) is a commonly-used first-line treatment for men with advanced prostate cancer. Although receiving ADT, the vast majority of patients eventually progress to a disease state known as castration-resistant prostate cancer (CRPC) [1]. CRPC is heterogenous, spanning from patients with a rising prostate-specific antigen (PSA) but no demonstrable metastases to patients with extensive metastases in visceral sites and/or bone [1]. A variety of markers, including circulating tumor cells (CTCs) and gene expression profiles, are being studied to identify subsets of metastatic CRPC (mCRPC) patients who have significantly different prognoses [2, 3]. CTCs have been associated with a poor prognosis in mCRPC patients [3, 4]. Further, a pooled analysis of five randomized clinical trials demonstrated CTC count to be a response measure in these patients [5].

Tumor metastasis depends not only on the intrinsic characteristics of the tumor cells, but also on the environment around the tumor [6]. The systematic inflammatory response is accompanied by changes in the relative levels of circulating white blood cells (WBCs), with concurrent increased neutrophils and decreased lymphocytes [7]. Walsh et al. first discovered the prognostic value of neutrophil-lymphocyte ratio (NLR) in pre-operative colorectal cancer patients [8]. Since then, there has been increasing evidence regarding the prognostic value of NLR in other solid tumors [9–12], including CRPC [13, 14].

CTCs have the capacity to bind and interact with non-malignant cells such as WBCs in the bloodstream, as seen in a recent study that demonstrated the metastatic potential of CTC-neutrophil clusters in both mouse models and patients with breast cancer [15]. The crosstalk between tumor cells and platelets also contributes to tumor metastasis by protecting CTCs from immune elimination [16]. However, no study has investigated whether these commonly available laboratory variables can further improve CTC-based prognostic stratification among mCRPC patients. To this end, we reviewed data from our prospective mCRPC cohort, analyzed baseline CTCs together with NLR and platelet-lymphocyte ratio (PLR), two hematological prognostic factors, and evaluated their joint impact on mCRPC survival.

## Patients and methods

### Study population

We prospectively recruited men with mCRPC who visited the Sidney Kimmel Cancer Center at Thomas Jefferson University Hospital starting in March 2018. All patients in this study had histologically confirmed prostate adenocarcinoma, progressive disease despite castration levels of serum testosterone (< 50 ng/dL), and radiographic metastases according to computed tomography (CT) or technetium-99 bone scan. Patients with other primary tumors were excluded. We reviewed medical charts to obtain baseline demographic data (e.g., age, race), clinical data (e.g., ECOG performance status [PS], treatments), and laboratory data (e.g., absolute neutrophil count, absolute lymphocyte count, platelet count, alkaline phosphatase [ALP], albumin [ALB], hemoglobin [HGB], lactate dehydrogenase [LDH], and PSA). NLR and PLR values were calculated accordingly. Blood samples were collected from each patient for CTC enumeration and baseline samples were obtained before initiation of a new therapy. Imaging tests during follow-up were conducted following the PCWG3 guideline [17]. This study was approved by the Institutional Review Board of Thomas Jefferson University. Each patient provided a written informed consent.

### CTC enumeration

Approximately 8–10 mL of whole blood were drawn into a 10 mL CellSave tube (Menarini Silicon Biosystems, Huntington Valley, Pennsylvania, USA), maintained at room temperature, and processed within 96 h of collection. CTC enumeration was conducted via the CellSearch System (Menarini Silicon Biosystems), which consists of the CellTracks Autoprep and the CellSearch CTC kit, to immunomagnetically enrich cells expressing the epithelial cell adhesion molecule. Cells were fluorescently labelled to identify the following: nuclei (DAPI), leukocytes with monoclonal antibodies specific for leukocytes (CD45), and epithelial cells (phycoerythrin-conjugated cytokeratins CK-8,18,19). CTCs were defined as nucleated cells lacking CD45 and expressing cytokeratin (CK+/DAPI+/CD45-) [3].

### Statistical analyses

The clinical outcomes analyzed in this study were progression-free survival (PFS) and overall survival (OS). PFS was defined as the time from the date of baseline blood draw to the date of radiologic progression (on CT scan: ≥20% enlargement in sum diameter of target lesions [Response Evaluation Criteria in Solid Tumors] [18]; on bone scan: ≥2 new bone lesions not caused by flare), symptomatic progression (worsening disease-related symptoms or new cancer-related complications), or death, whichever occurred first [19]. OS was defined as the time from the date of baseline blood draw to the date of death from any cause. The patients without an endpoint event at the last follow-up visit were censored. The cutoff values of NLR and PLR for dichotomizing patients into high- and low-level groups were determined using receiver operating characteristic curve (ROC) analysis. We plotted survival curves using the Kaplan-Meier estimator and compared survival differences using the log-rank test. Time-dependent ROC analyses were used to compare the performance between a CTC model with or without NLR/PLR, and to explore the discriminatory ability over time. Associations of CTC (absence/presence), NLR (high/low), and PLR (high/low), individually and jointly, with PFS or OS were evaluated using hazard ratios (HRs) with 95% confidence intervals (CIs) by univariate and multivariate Cox proportional hazards models. Only variables that were significantly associated with outcomes in the univariate analyses were controlled in the multivariate model. The proportional hazards assumption was validated using the test based on Schoenfeld residuals. SAS (Version 9.4, SAS Institute, Cary, NC) and STATA (Version 11.0, STATA Corp., College Station, TX) software packages were used for the analyses conducted in this study. All *P* values were 2-sided, with a *P* < 0.05 considered the threshold for statistical significance.

## Results

### Patient characteristics

Sixty-three mCRPC patients with both CTC enumeration results and NLR/PLR values were included in this analysis. Among the patients with a median age of 70 years (range 52 to 93), 60 (95.2%) patients had metastasis to bone, and 12 (19%) patients had visceral metastases. Prior to baseline CTC measurement, 29 (46%) and 25 (39.7%) patients were ever treated with androgen receptor signaling inhibitors (ARSi), such as abiraterone acetate and enzalutamide, and cytotoxic chemotherapy, respectively. There were 47 (74.6%) and 17 (27%) patients receiving ARSi and chemotherapy, respectively, since enrollment. During a median follow-up period of 17.6 months (interquartile range [IQR]: 10.3–20.6), 23 (36.5%) patients died. Details of patient characteristics are summarized in Table 1.

**Table 1:** Patient characteristics (*N* = 63)

Variables	N (%)
Age (year), median (range)	70.9 (52.7–93.0)
Race	
White	49 (77.8)
Black	11 (17.5)
Other	3 (4.8)
Gleason score at diagnosis	
6	3 (4.8)
7	15 (23.8)
8	10 (15.9)
9	26 (41.3)
10	4 (6.4)
Unknown	5 (7.9)
ECOG performance status	
0	25 (39.7)
1	28 (44.4)
2	8 (12.7)
3	1 (1.6)
Unknown	1 (1.6)
Bone metastasis	
No	3 (4.8)
Yes	60 (95.2)
Visceral metastasis	
No	51 (81.0)
Yes	12 (19.0)
Previous ARSi therapy	
No	34 (54.0)
Yes	29 (46.0)
Previous chemotherapy	
No	38 (60.3)
Yes	25 (39.7)
ARSi therapy after blood draw	
No	16 (25.4)
Yes	47 (74.6)
Cytotoxic therapy after blood draw	
No	46 (73.0)
Yes	17 (27.0)
Absolute neutrophil (B/L), median (range)	4.2 (1.0–15.4)
Absolute lymphocyte (B/L), median (range)	1.0 (0.3–10.4)
Platelet (B/L), median (range)	214 (73–513)
Neutrophil-to-lymphocyte ratio, median (range)	3.7 (0.3–20.6)
Platelet-to-lymphocyte ratio, median (range)	200 (12.9–1126.9)
Prostate-specific antigen (ng/ml), median (range)	8.8 (0.1–1169.0)
Hemoglobin (g/dL), median (range)	12.1 (7.4–14.6)
Alkaline phosphatase (IU/L), median (range)	86 (36–1709)
Albumin (g/dL), median (range)	4.1 (2.7–4.7)
Lactate dehydrogenase (IU/L), median (range)^a^	212 (149–560)
Vital status	
Alive	40 (63.5)
Dead	23 (36.5)

*ECOG* Eastern Cooperative Oncology Group; *ARSi* androgen receptor signaling inhibitor

^a^: data were available from 24 patients

### Association between CTC and clinical outcomes

**Fig. 1:**
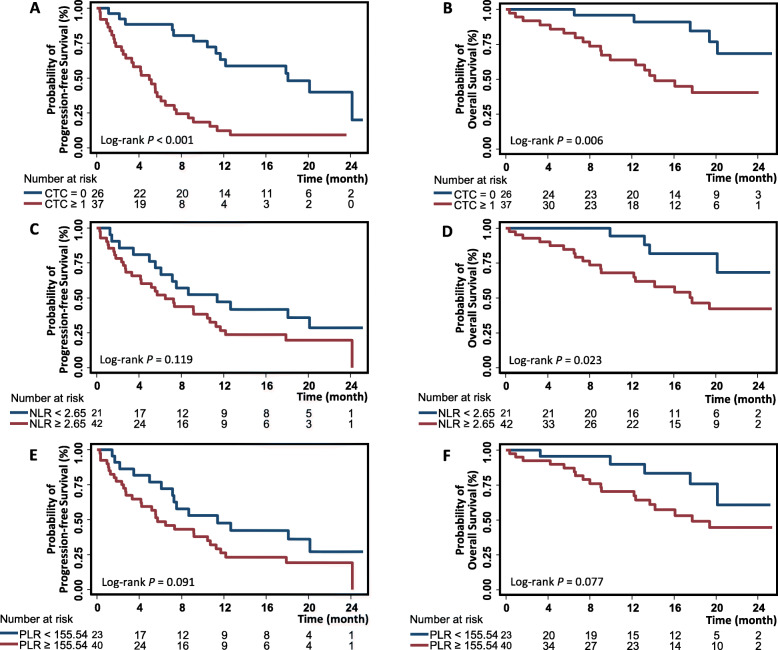
Kaplan-Meier survival plots of mCRPC patients. The survival differences were compared: (1) between patients without CTC and those with CTCs (≥1) (**A** for PFS analysis and **B** for OS analysis); (2) between patients with low levels of NLR and those with high levels of NLR (**C** for PFS analysis and **D** for OS analysis); (3) between patients with low levels of PLR and those with high levels of PLR (**E** for PFS analysis and **F** for OS analysis). Survival differences were compared with the log rank test, with *P* < 0.05 denoting significance. mCRPC: metastatic castration-resistant prostate cancer; CTC: circulating tumor cell; NLR: neutrophil-lymphocyte ratio; PLR: platelet-lymphocyte ratio; PFS: progression-free survival; OS: overall survival

CTCs were detected in 37 (58.7%) of 63 baseline samples, and the median CTC count was 3 (IQR: 1–17). Among mCRPC patients with CTCs, 16 had five or more CTCs, a high CTC count that has been associated with worse clinical outcomes in previous studies [3–5]. We found that, compared to patients without CTCs, patients with CTCs (≥1) had lower PFS (5.0 months vs. 18.1 months, log-rank *P* < 0.001) (Table 2, Fig. 1A). Compared to patients without CTCs, patients with CTCs experienced a 4.02-fold risk of progression (HR 4.02, 95% CI 2.05 to 7.86, Table 2). Similarly**,** the presence of CTCs was associated with shorter OS (14.2 months vs. not reached [NR], log-rank *P* = 0.006), and patients with CTCs had a 3.72-fold risk of death (HR 3.72, 95% CI 1.37 to 10.06) (Table 2, Fig. 1B). We conducted the same analyses using the widely accepted cut-off of 5 CTCs and obtained similar results (Figure S1).

**Table 2:** Univariate analysis of associations with clinical outcomes

Variables	Total	Event	Median survival (mo)	Log-rank ***P***	HR (95% CI)	***P***
**Association with PFS**						
CTC						
0	26	14	18.1	**< 0.001**	Ref.	
≥ 1	37	31	5.0		4.02 (2.05–7.86)	**< 0.001**
NLR						
< 2.65	21	14	11.4	0.119	Ref.	
≥ 2.65	42	31	6.5		1.65 (0.87–3.12)	0.123
PLR						
< 155.54	23	14	11.4	0.091	Ref.	
≥ 155.54	40	31	5.7		1.72 (0.91–3.24)	0.095
Risk group						
CTC = 0 and NLR < 2.65	9	4	20.1	**< 0.001**	Ref.	
CTC = 0 and NLR ≥ 2.65	17	10	12.2		1.92 (0.59–6.29)	0.279
CTC ≥ 1 and NLR < 2.65	12	10	6.1		Ref.	
CTC ≥ 1 and NLR ≥ 2.65	25	21	3.3		2.12 (0.97–4.61)	0.059
CTC = 0 and PLR < 155.54	11	5	20.1	**< 0.001**	Ref.	
CTC = 0 and PLR ≥ 155.54	15	9	17.9		1.42 (0.47–4.29)	0.531
CTC ≥ 1 and PLR < 155.54	12	9	7.5		Ref.	
CTC ≥ 1 and PLR ≥ 155.54	25	22	4.1		2.27 (1.02–5.08)	**0.046**
**Association with OS**						
CTC						
0	26	5	NR	**0.006**	Ref.	
≥ 1	37	18	14.2		3.72 (1.37–10.06)	**0.010**
NLR						
< 2.65	21	4	NR	**0.023**	Ref.	
≥ 2.65	42	19	17.7		3.27 (1.11–9.63)	**0.031**
PLR						
< 155.54	23	5	NR	0.077	Ref.	
≥ 155.54	40	18	17.7		2.38 (0.88–6.43)	0.086
Risk group						
CTC = 0 and NLR < 2.65	9	1	NR	**0.002**	Ref.	
CTC = 0 and NLR ≥ 2.65	17	4	NR		3.12 (0.34–28.34)	0.313
CTC ≥ 1 and NLR < 2.65	12	3	NR		Ref.	
CTC ≥ 1 and NLR ≥ 2.65	25	15	9.1		3.79 (1.09–13.13)	**0.036**
CTC = 0 and PLR < 155.54	11	2	NR	**0.007**	Ref.	
CTC = 0 and PLR ≥ 155.54	15	3	NR		1.19 (0.20–7.18)	0.846
CTC ≥ 1 and PLR < 155.54	12	3	NR		Ref.	
CTC ≥ 1 and PLR ≥ 155.54	25	15	13.7		2.85 (0.82–9.86)	0.098

*CTC* circulating tumor cell; *NLR* neutrophil-to-lymphocyte ratio; *PLR* platelet-to-lymphocyte ratio; *PFS* progression free survival; *OS* overall survival; *NR* not reached; *HR* hazard ratio; *CI* confidence interval

### Association between NLR and clinical outcomes

According to the cut-off (2.65) determined using the ROC analysis, patients were classified into two groups: the low-NLR (*n* = 21) and the high-NLR group (*n* = 42). The high-NLR group exhibited lower PFS (6.5 months vs. 11.4 months in low-NLR group), although the difference in PFS did not reach significance (log-rank *P* = 0.119) (Table 2, Fig. 1C). There was a statistically significant difference in OS between the high-NLR and low-NLR groups (17.7 months vs. NR, log-rank *P* = 0.023, Fig. 1D). A 3.27-fold risk of death was observed in the high-NLR group compared to the low-NLR group (HR 3.27, 95% CI 1.11 to 9.63) (Table 2).

### Association between PLR and clinical outcomes

Using the cutoff of PLR (155.54) to classify the patients into two groups, we obtained the low-PLR (*n* = 23) and the high-PLR groups (*n* = 40). The high-PLR group showed a trend towards earlier progression (median PFS of 5.7 months vs. 11.4 months in the low-PLR group) and lower OS (17.7 months vs. NR in the low-PLR group) (Table 2). However, neither of the survival metrics between the two groups reached statistical significance (log-rank *P* = 0.091 and 0.077, respectively) (Fig. 1E, F). The univariate Cox analyses of associations between PLR and outcomes yielded similar results (Table 2).

### Joint effect of CTC and NLR on clinical outcomes

**Fig. 2:**
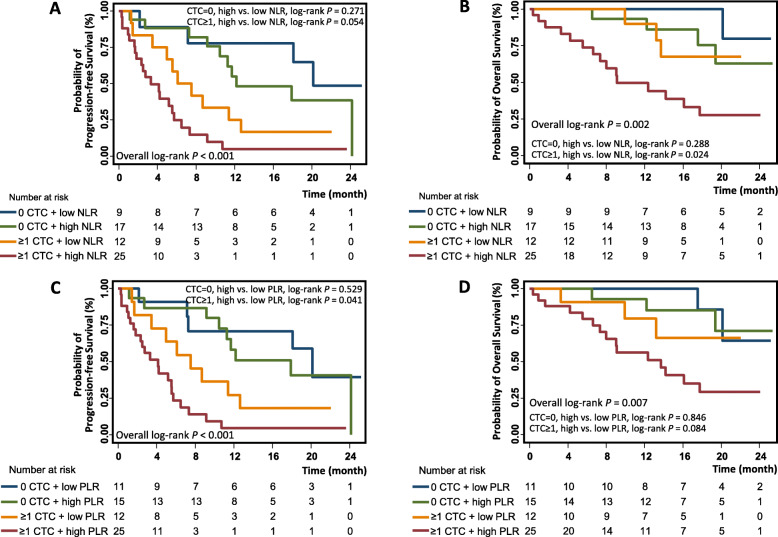
Kaplan-Meier survival plots of mCRPC patients. The survival differences were compared among four risk groups which was stratified according to the status of CTCs (absence/presence) and NLR level (low/high) (**A** for PFS analysis and **B** for OS analysis), or according to the status of CTCs (absence/presence) and PLR level (low/high) (**C** for PFS analysis and **D** for OS analysis). Survival differences were compared with the log rank test, with *P* < 0.05 denoting significance. mCRPC: metastatic castration-resistant prostate cancer; CTC: circulating tumor cell; NLR: neutrophil-lymphocyte ratio; PLR: platelet-lymphocyte ratio; PFS: progression-free survival; OS: overall survival

To analyze the joint effects of CTC and NLR on clinical outcomes, we subdivided the study population into four groups: Group 1, CTC = 0 and NLR < 2.65 (*n* = 9); Group 2, CTC = 0 and NLR ≥ 2.65 (*n* = 17); Group 3, CTC ≥ 1 and NLR < 2.65 (*n* = 12); and Group 4, CTC ≥ 1 and NLR ≥ 2.65 (*n* = 25) (Table 2). Comparing the PFS with the log-rank test, we found that the median time to progression decreased from Group 1 to Group 4 (20.1, 12.2, 6.1, and 3.3 months, respectively, log-rank *P* < 0.001) (Table 2, Fig. 2A). However, the differences in PFS between Group 1 and Group 2 (log-rank *P* = 0.271), or between Group 3 and Group 4 (log-rank *P* = 0.054) were not statistically significant. We then conducted similar analyses for OS, and found that patients in Group 4 had the shortest median OS of 9.1 months (Fig. 2B). Moreover, among the patients with detectable CTCs (Groups 3 and 4), those with a high NLR had significantly shorter OS than those with a low NLR (log-rank *P* = 0.024, Fig. 2B), indicating a further prognostic stratification using NLR. It should be noted that, among the patients without CTCs, the difference in OS between high and low NLR groups was not statistically significant (log-rank *P* = 0.288), thus the stratification effect of NLR only existed in the patients whose tumor cells presented in circulation. Univariate Cox analysis also showed that, compared to patients with both CTCs and low NLRs, those with both CTCs and high levels of NLR had a 3.79-fold risk of death (HR 3.79, 95% CI 1.09–13.13) (Table 2).

### Joint effect of CTC and PLR on clinical outcomes

Similarly, we classified patients into four groups based on their CTC counts and PLR values: CTC = 0 and PLR < 155.54 (*n* = 11); CTC = 0 and PLR ≥ 155.54 (*n* = 15); CTC ≥ 1 and PLR < 155.54 (*n* = 12); and CTC ≥ 1 and PLR ≥ 155.54 (*n* = 25). The overall differences in PFS and OS among the four groups were both significant (log-rank *P* < 0.001 and *P* = 0.007, respectively, Fig. 2C and D). Furthermore, among the patients having ≥1 CTCs, those with high PLR values had shorter PFS and OS than those with low PLR values, and the survival differences were significant for PFS analysis (log-rank *P* = 0.041). Similar results were obtained from univariate Cox analyses. Compared to the patients with CTCs and low PLRs, those with CTCs and high PLRs had significantly increased risk for PFS (HR 2.27, *P* = 0.046) (Table 2).

### Evaluation of predictive power of a model combining NLR or PLR

**Fig. 3:**
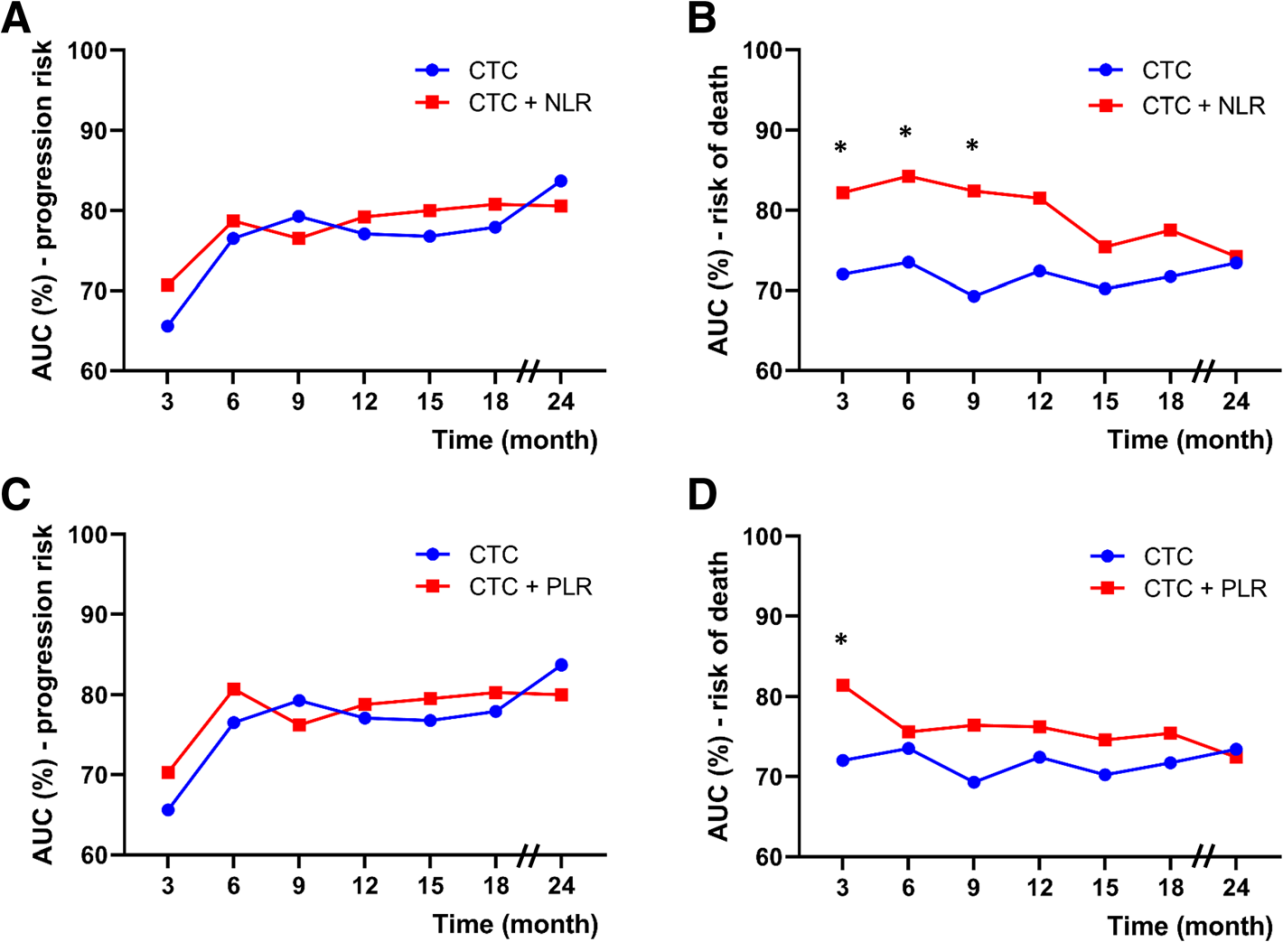
Time-dependent ROC analyses for survival prediction models. AUCs (%) in predicting progression or death risk of mCRPC patients over time were estimated and compared between a CTC only model and a model in combination of CTC and NLR (**A** for progression risk and **B** for death risk), or a model in combination of CTC and PLR (**C** for progression risk and D for death risk). ROC: receiver operating characteristic; AUC: area under the curve; mCRPC: metastatic castration-resistant prostate cancer; CTC: circulating tumor cell; NLR: neutrophil-lymphocyte ratio; PLR: platelet-lymphocyte ratio. Star indicates a *P* value of < 0.001 when comparing the AUC derived from a CTC only model and that from a combination model

To demonstrate whether NLR or PLR provided additional prognostic value, the performance between a CTC model with and a model without NLR/PLR were estimated and compared by time-dependent ROC analyses. We found that the performance of a model in combination of CTC and NLR was higher than a CTC only model in predicting death risk (AUC: 82.2% vs. 72.0% at 3 m, *P* < 0.001; 84.3% vs. 73.5% at 6 m, *P* < 0.001; 82.4% vs. 69.3% at 9 m, *P* < 0.001; 81.5% vs. 72.4% at 12 m, *P* = 0.061; 77.5% vs. 71.7% at 18 m, *P* = 0.271; 74.2% vs. 73.4% at 24 m, *P* = 0.893, Fig. 3). Thus, NLR added prognostic value to that offered by CTC alone, although the discriminatory power decreased over time. No significant result was obtained in other combination models, except for a significantly higher performance in predicting death risk at 3 m using a CTC plus PLR model than a CTC only model (AUC 81.4% vs. 72%, *P* < 0.001).

### Multivariate analysis of joint associations with clinical outcomes

**Fig. 4:**
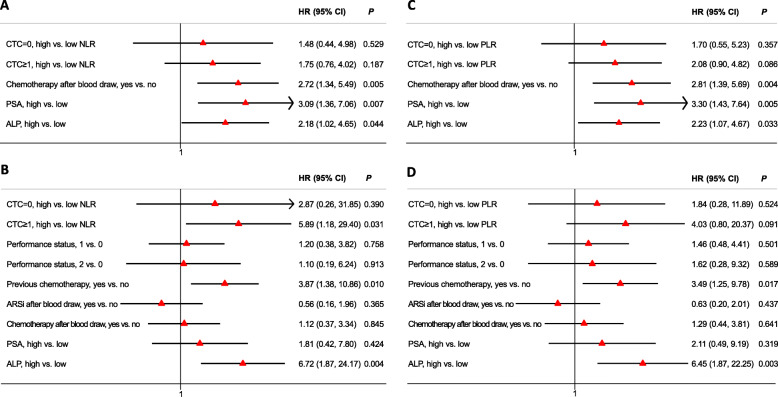
Multivariate analyses of associations with clinical outcomes in mCRPC patients. Risk groups were defined according to CTC counts and NLR values (A for PFS analysis and B for OS analysis), or defined according to CTC counts and PLR values (C for PFS analysis and D for OS analysis). Covariates applied in the model for PFS included chemotherapy after blood draw (yes vs. low), PSA (high vs. low), and ALP (high vs. low). For the OS model, the following covariates were also applied: ECOG performance status (1 vs. 0, ≥2 vs. 0), previous chemotherapy (yes vs. no), and ARSi after blood draw (yes vs. no). mCRPC: metastatic castration-resistant prostate cancer; ARSi: androgen receptor signaling inhibitor; CTC: circulating tumor cell; NLR: neutrophil-lymphocyte ratio; PLR: platelet-lymphocyte ratio; PSA: prostate-specific antigen; ALP: alkaline phosphatase; PFS: progression-free survival; OS: overall survival; HR, hazard ratio; CI: confidence interval

The univariate analyses suggested that NLR and PLR might provide additional prognostic information among patients with CTCs. To find out whether the identified associations were independent of clinical confounders, we developed multivariate Cox models by combining confounding variables such as ECOG PS identified from univariate analyses (Table S1). Figure 4 shows the results from multivariate Cox analyses. Among the patients with one or more CTCs, the association between NLR and OS remained statistically significant (HR 5.89, 95% CI 1.18 to 29.40, *P* = 0.031) after adjusting covariates (PS, treatments, PSA, and ALP) (Fig. 4B), further confirming the prognostic stratification from NLR in addition to CTC enumeration alone. Other unfavorable prognostic factors included previously receiving chemotherapy, high PSA, and high ALP values (Fig. 4).

## Discussion

The vast majority of prostate-cancer specific deaths occur in the setting of castration-resistant disease. Validated prognostic biomarkers can be used to more accurately inform physicians and patients and to assist in the development of life-prolonging treatment plans. Multiple hematological biomarkers have been associated with prognosis of CRPC, such as PSA [20–23], ALP [24, 25], LDH [23, 26], and HGB [23, 27]. These routine laboratory parameters, although imperfect, are always combined into statistical models to predict mCRPC outcomes [27, 28].

CTCs have promising prognostic and predictive value in cancers including CRPC [3, 29]. In a recent phase III clinical trial of abiraterone acetate plus prednisone versus prednisone alone in patients with mCRPC, a biomarker panel containing CTC number and LDH level was shown to be a surrogate for OS at the individual-patient level [30]. NLR and PLR are inflammatory parameters that also confer poor outcomes in mCRPC [13, 14, 31, 32] and are easily available from routine complete blood counts and more stable compared to absolute counts [33]. However, the additional prognostic value of NLR and PLR has never been evaluated in the context of CTCs. Based on the data from our mCRPC cohort, we observed unfavorable outcomes in the patients with CTCs (≥1 or ≥ 5) and high levels of NLR or PLR. Importantly, we found that NLR could further classify risk of death among those with CTCs, but not among those without CTCs. The performance in predicting risk of death was improved by adding NLR to a CTC model, although the discriminatory accuracy decreased over time. Moreover, the joint association was independent of clinical confounders. These results suggest a new avenue for improving risk-stratified management of mCRPC.

The shedding of tumor cells into circulation is a necessary, but not sufficient condition for the formation of metastases [30, 34]. The interplay between tumor cells and host microenvironment plays an important role in tumor cell dissemination. Chronic inflammation is a classic and prevalent example of ongoing perturbation within the microenvironment. Sustained inflammation contributes to proliferation and survival of malignant cells, angiogenesis, metastasis, and subversion of adaptive immunity [31]. Moreover, cancer-associated systemic inflammation is likely to interfere with effective treatments due to the interaction between the systemic inflammation and the inhibition of cytochrome P450 [31], which is especially important for mCRPC patients because of the widely used first-line agent abiraterone acetate, a CYP17 inhibitor. Neutrophil extracellular traps, which are neutrophil-derived DNA webs released in response to inflammatory cues, have been shown to sequester CTCs and promote metastases [35]. Furthermore, CTC-neutrophil clusters have been detected in the blood of metastatic breast cancer patients, and the association between neutrophils and CTCs drives cell cycle progression within the bloodstream and expands the metastatic potential of CTCs [15]. Low absolute lymphocyte counts have been associated with a generalized state of immunosuppression in several types of cancer [13]. Both increased neutrophil-dependent systemic inflammatory response and a lower lymphocyte-mediated antitumor immune response will lead to an elevated NLR [14]. Therefore, the individual and joint associations of CTC and NLR with OS identified in this study is biologically plausible, although the exact mechanisms underlying their joint impact need to be further elucidated.

Our study focused on mCRPC patients, which ensures a more homogenous study population. By integrating NLR and PLR - two inexpensive and convenient hematological parameters from routine blood tests - into prognostic models, we determined that NLR provides additional prognostic value in patients with CTCs for improved risk stratification and optimal management. The major limitations of this study included small sample size, lack of independent validation, and not adjusting important confounders such as LDH due to incomplete data. In addition, although enrolled patients were relatively homogeneous in terms of tumor stages and the state of castration resistance, the therapies they received were still heterogeneous, given the fact that a portion of patients were previously treated; however, it was infeasible to conduct a regimen-based subgroup analysis due to insufficient power.

## Conclusion

Among mCRPC patients with detectable CTCs, a high NLR is a negative prognostic factor for overall survival. The additional prognostic stratification of NLR needs to be further tested in future large prospective studies.

## Supplementary Information

**Additional file 1: Table S1.** Univariate analysis of associations with clinical outcomes. **Figure S1.** Kaplan-Meier survival plots of mCRPC patients. The survival differences were compared between patients with ≥5 CTCs and those with < 5 CTCs (A for PFS analysis and B for OS analysis).

## Data Availability

The datasets used and/or analyzed during the current study are available from the corresponding author on reasonable request.

## References

[1] Kirby M, Hirst C, Crawford E (2011). Characterising the castration-resistant prostate cancer population: a systematic review. Int J Clin Pract.

[2] Eggener SE, Rumble RB, Armstrong AJ, Morgan TM, Crispino T, Cornford P, van der Kwast T, Grignon DJ, Rai AJ, Agarwal N (2020). Molecular biomarkers in localized prostate cancer: ASCO guideline. J Clin Oncol.

[3] De Bono JS, Scher HI, Montgomery RB, Parker C, Miller MC, Tissing H, Doyle GV, Terstappen LW, Pienta KJ, Raghavan D (2008). Circulating tumor cells predict survival benefit from treatment in metastatic castration-resistant prostate cancer. Clin Cancer Res.

[4] Olmos D, Arkenau H-T, Ang J, Ledaki I, Attard G, Carden C, Reid A, A'Hern R, Fong P, Oomen N (2009). Circulating tumour cell (CTC) counts as intermediate end points in castration-resistant prostate cancer (CRPC): a single-Centre experience. Ann Oncol.

[5] Heller G, McCormack R, Kheoh T, Molina A, Smith MR, Dreicer R, Saad F, de Wit R, Aftab DT, Hirmand M, Limon A, Fizazi K, Fleisher M, de Bono JS, Scher HI (2018). Circulating tumor cell number as a response measure of prolonged survival for metastatic castration-resistant prostate Cancer: a comparison with prostate-specific antigen across five randomized phase III clinical trials. J Clin Oncol.

[6] Gajewski TF, Schreiber H, Fu Y-X (2013). Innate and adaptive immune cells in the tumor microenvironment. Nat Immunol.

[7] Zahorec R (2001). Ratio of neutrophil to lymphocyte counts-rapid and simple parameter of systemic inflammation and stress in critically ill. Bratislavske lekarske listy.

[8] Walsh S, Cook E, Goulder F, Justin T, Keeling N (2005). Neutrophil-lymphocyte ratio as a prognostic factor in colorectal cancer. J Surg Oncol.

[9] Tham T, Bardash Y, Herman SW, Costantino PD (2018). Neutrophil-to-lymphocyte ratio as a prognostic indicator in head and neck cancer: a systematic review and meta-analysis. Head Neck.

[10] Li MX, Liu XM, Zhang XF, Zhang JF, Wang WL, Zhu Y, Dong J, Cheng JW, Liu ZW, Ma L (2014). Prognostic role of neutrophil-to-lymphocyte ratio in colorectal cancer: a systematic review and meta-analysis. Int J Cancer.

[11] Gu X, Gao X, Li X, Qi X, Ma M, Qin S, Yu H, Sun S, Zhou D, Wang W (2016). Prognostic significance of neutrophil-to-lymphocyte ratio in prostate cancer: evidence from 16,266 patients. Sci Rep.

[12] Templeton AJ, McNamara MG, Šeruga B, Vera-Badillo FE, Aneja P, Ocaña A, Leibowitz-Amit R, Sonpavde G, Knox JJ, Tran B (2014). Prognostic role of neutrophil-to-lymphocyte ratio in solid tumors: a systematic review and meta-analysis. J Natl Cancer Inst.

[13] Nuhn P, Vaghasia AM, Goyal J, Zhou XC, Carducci MA, Eisenberger MA, Antonarakis ES (2014). Association of pretreatment neutrophil-to-lymphocyte ratio (NLR) and overall survival (OS) in patients with metastatic castration-resistant prostate cancer (mCRPC) treated with first-line docetaxel. BJU Int.

[14] Wang Z, Peng S, Xie H, Guo L, Jiang N, Shang Z, Niu Y (2018). Neutrophil-lymphocyte ratio is a predictor of prognosis in patients with castration-resistant prostate cancer: a meta-analysis. Cancer Manag Res.

[15] Szczerba BM, Castro-Giner F, Vetter M, Krol I, Gkountela S, Landin J, Scheidmann MC, Donato C, Scherrer R, Singer J (2019). Neutrophils escort circulating tumour cells to enable cell cycle progression. Nature.

[16] Kanikarla-Marie P, Lam M, Menter DG, Kopetz S (2017). Platelets, circulating tumor cells, and the circulome. Cancer Metastasis Rev.

[17] Scher HI, Morris MJ, Stadler WM, Higano C, Basch E, Fizazi K, Antonarakis ES, Beer TM, Carducci MA, Chi KN, Corn PG, de Bono JS, Dreicer R, George DJ, Heath EI, Hussain M, Kelly WK, Liu G, Logothetis C, Nanus D, Stein MN, Rathkopf DE, Slovin SF, Ryan CJ, Sartor O, Small EJ, Smith MR, Sternberg CN, Taplin ME, Wilding G, Nelson PS, Schwartz LH, Halabi S, Kantoff PW, Armstrong AJ, Prostate Cancer Clinical Trials Working Group 3 (2016). Trial design and objectives for castration-resistant prostate Cancer: updated recommendations from the prostate Cancer clinical trials working group 3. J Clin Oncol.

[18] Eisenhauer EA, Therasse P, Bogaerts J, Schwartz LH, Sargent D, Ford R, Dancey J, Arbuck S, Gwyther S, Mooney M, Rubinstein L, Shankar L, Dodd L, Kaplan R, Lacombe D, Verweij J (2009). New response evaluation criteria in solid tumours: revised RECIST guideline (version 1.1). Eur J Cancer.

[19] Antonarakis ES, Lu C, Luber B, Wang H, Chen Y, Zhu Y, Silberstein JL, Taylor MN, Maughan BL, Denmeade SR, Pienta KJ, Paller CJ, Carducci MA, Eisenberger MA, Luo J (2017). Clinical significance of androgen receptor splice Variant-7 mRNA detection in circulating tumor cells of men with metastatic castration-resistant prostate Cancer treated with first- and second-line Abiraterone and enzalutamide. J Clin Oncol.

[20] Arlen PM, Bianco F, Dahut WL, D'Amico A, Figg WD, Freedland SJ, Gulley JL, Kantoff PW, Kattan MW, Lee A, Regan MM, Sartor O, Prostate Specific Antigen Working Group (2008). Prostate specific antigen working group guidelines on prostate specific antigen doubling time. J Urol.

[21] Fleming MT, Morris MJ, Heller G, Scher HI (2006). Post-therapy changes in PSA as an outcome measure in prostate cancer clinical trials. Nat Clin Pract Oncol.

[22] Scher HI, Morris MJ, Basch E, Heller G (2011). End points and outcomes in castration-resistant prostate cancer: from clinical trials to clinical practice. J Clin Oncol.

[23] Armstrong AJ, Eisenberger MA, Halabi S, Oudard S, Nanus DM, Petrylak DP, Sartor AO, Scher HI (2012). Biomarkers in the management and treatment of men with metastatic castration-resistant prostate cancer. Eur Urol.

[24] Sonpavde G, Pond GR, Berry WR, de Wit R, Armstrong AJ, Eisenberger MA, Tannock IF (2012). Serum alkaline phosphatase changes predict survival independent of PSA changes in men with castration-resistant prostate cancer and bone metastasis receiving chemotherapy. Urol Oncol.

[25] Heinrich D, Bruland Ø, Guise TA, Suzuki H, Sartor O (2018). Alkaline phosphatase in metastatic castration-resistant prostate cancer: reassessment of an older biomarker. Future Oncol.

[26] Hiew K, Hart CA, Ali A, Elliott T, Ramani V, Sangar V, Lau M, Maddineni S, Brown M, Clarke N (2019). Primary mutational landscape linked with pre-docetaxel lactate dehydrogenase levels predicts docetaxel response in metastatic castrate-resistant prostate Cancer. Eur Urol Focus.

[27] Armstrong AJ, Garrett-Mayer ES, Yang YC, de Wit R, Tannock IF, Eisenberger M (2007). A contemporary prognostic nomogram for men with hormone-refractory metastatic prostate cancer: a TAX327 study analysis. Clin Cancer Res.

[28] Halabi S, Lin CY, Kelly WK, Fizazi KS, Moul JW, Kaplan EB, Morris MJ, Small EJ (2014). Updated prognostic model for predicting overall survival in first-line chemotherapy for patients with metastatic castration-resistant prostate cancer. J Clin Oncol.

[29] Goodman OB, Fink LM, Symanowski JT, Wong B, Grobaski B, Pomerantz D, Ma Y, Ward DC, Vogelzang NJ (2009). Circulating tumor cells in patients with castration-resistant prostate cancer baseline values and correlation with prognostic factors. Cancer Epidemiol Biomark Prev.

[30] Scher HI, Heller G, Molina A, Attard G, Danila DC, Jia X, Peng W, Sandhu SK, Olmos D, Riisnaes R, McCormack R, Burzykowski T, Kheoh T, Fleisher M, Buyse M, de Bono JS (2015). Circulating tumor cell biomarker panel as an individual-level surrogate for survival in metastatic castration-resistant prostate cancer. J Clin Oncol.

[31] Lozano Martínez AJ, Moreno Cano R, Escobar Páramo S, Salguero Aguilar R, Gonzalez Billalabeitia E, García Fernández R, De La Fuente MI, Romero Borque A, Porras Martínez M, Lopez Soler F (2017). Platelet-lymphocyte and neutrophil-lymphocyte ratios are prognostic but not predictive of response to abiraterone acetate in metastatic castration-resistant prostate cancer. Clin Transl Oncol.

[32] Donate-Moreno MJ, Lorenzo-Sánchez MV, Díaz de Mera-Sánchez Migallón I, Herraiz-Raya L, Esper-Rueda JA, Legido-Gómez O, et al. Inflammatory markers as prognostic factors in metastatic castration-resistant prostate cancer. Actas Urol Esp. 2020;44(10):692–700. 10.1016/j.acuroe.2020.11.009. 10.1016/j.acuro.2020.08.001 33010988

[33] Azab B, Bhatt VR, Phookan J, Murukutla S, Kohn N, Terjanian T, Widmann WD (2012). Usefulness of the neutrophil-to-lymphocyte ratio in predicting short- and long-term mortality in breast cancer patients. Ann Surg Oncol.

[34] Pantel K, Brakenhoff RH, Brandt B (2008). Detection, clinical relevance and specific biological properties of disseminating tumour cells. Nat Rev Cancer.

[35] Cools-Lartigue J, Spicer J, McDonald B, Gowing S, Chow S, Giannias B, Bourdeau F, Kubes P, Ferri L (2013). Neutrophil extracellular traps sequester circulating tumor cells and promote metastasis. J Clin Invest.

