# Supplementary material for: Integration of circulating tumor cell and neutrophil-lymphocyte ratio to identify high-risk metastatic castration-resistant prostate cancer patients
Source: BMC Cancer. 2021 Jun 2;21:655. doi: 10.1186/s12885-021-08405-3 (PMC8170812; doi:10.1186/s12885-021-08405-3)

**Integration of circulating tumor cell and neutrophil-lymphocyte ratio to identify high-risk metastatic castration-resistant prostate cancer patients**

Weelic Chong^1†^, Zhenchao Zhang^1†^, Rui Luo^1^, Jian Gu^2^, Jianqing Lin^3^, Qiang Wei^4^, Bingshan Li^4^, Ronald Myers^1^, Grace Lu-Yao^1^, William Kevin Kelly^1^, Chun Wang^1*^, Hushan Yang^1*^

^1^ Department of Medical Oncology, Sidney Kimmel Cancer Center, Thomas Jefferson University, Philadelphia, PA 19107, USA

^2^ Department of Epidemiology, MD Anderson Cancer Center, Houston, TX 77030, USA

^3^ Department of Medicine, GW Cancer Center, George Washington University, Washington, DC 20037, USA

^4^ Department of Molecular Physiology and Biophysics, Vanderbilt University, Nashville, TN 37235, USA

**Table S1.** Univariate analysis of associations with clinical outcomes

| **Variables** | **N** | **Progression-free survival** | |  | **Overall survival** | |
| --- | --- | --- | --- | --- | --- | --- |
|  |  | **HR (95% CI)** | ***P*** |  | **HR (95% CI)** | ***P*** |
| Age (year) | 63 | 0.99 (0.96-1.03) | 0.609 |  | 1.00 (0.95-1.05) | 0.844 |
| <70.88 | 32 | Ref. |  |  | Ref. |  |
| ≥70.88 | 31 | 0.72 (0.40-1.30) | 0.274 |  | 1.11 (0.49-2.51) | 0.809 |
| Race |  |  |  |  |  |  |
| White | 49 | Ref. |  |  | Ref. |  |
| Black | 11 | 0.84 (0.39-1.82) | 0.654 |  | 0.59 (0.18-1.98) | 0.393 |
| Other | 3 | 1.01 (0.24-4.21) | 0.994 |  | - | 0.993 |
| Gleason score at diagnosis |  |  |  |  |  |  |
| ≤7 | 18 | Ref. |  |  | Ref. |  |
| >7 | 40 | 1.06 (0.54-2.07) | 0.875 |  | 0.84 (0.34-2.09) | 0.709 |
| ECOG performance status |  |  |  |  |  |  |
| 0 | 25 | Ref. |  |  | Ref. |  |
| 1 | 28 | 1.09 (0.57-2.09) | 0.787 |  | 2.17 (0.76-6.17) | 0.147 |
| ≥2 | 9 | 1.81 (0.74-4.41) | 0.191 |  | 10.28 (2.95-35.87) | **<0.001** |
| Bone metastasis |  |  |  |  |  |  |
| No | 3 | Ref. |  |  | Ref. |  |
| Yes | 60 | 4.34 (0.58-32.59) | 0.154 |  | - | 0.992 |
| Visceral metastasis |  |  |  |  |  |  |
| No | 51 | Ref. |  |  | Ref. |  |
| Yes | 12 | 0.75 (0.35-1.61) | 0.458 |  | 1.14 (0.45-2.92) | 0.782 |
| Previous ARSi therapy |  |  |  |  |  |  |
| No | 34 | Ref. |  |  | Ref. |  |
| Yes | 29 | 1.17 (0.65-2.11) | 0.595 |  | 1.82 (0.79-4.16) | 0.158 |
| Previous chemotherapy |  |  |  |  |  |  |
| No | 38 | Ref. |  |  | Ref. |  |
| Yes | 25 | 1.77 (0.97-3.24) | 0.062 |  | 2.79 (1.22-6.39) | **0.015** |
| ARSi therapy after blood draw |  |  |  |  |  |  |
| No | 16 | Ref. |  |  | Ref. |  |
| Yes | 47 | 0.74 (0.39-1.42) | 0.367 |  | 0.40 (0.17-0.97) | **0.043** |
| Cytotoxic therapy after blood draw |  |  |  |  |  |  |
| No | 46 | Ref. |  |  | Ref. |  |
| Yes | 17 | 4.17 (2.14-8.13) | **<0.001** |  | 3.36 (1.47-7.69) | **0.004** |
| Prostate-specific antigen (ng/ml) |  |  |  |  |  |  |
| ≤4 | 22 | Ref. |  |  | Ref. |  |
| >4 | 41 | 4.51 (2.19-9.29) | **<0.001** |  | 4.50 (1.52-13.36) | **0.007** |
| Hemoglobin (g/dL) |  |  |  |  |  |  |
| ≥14 | 8 | Ref. |  |  | Ref. |  |
| <14 | 55 | 1.14 (0.48-2.70) | 0.768 |  | 4.34 (0.59-32.25) | 0.151 |
| Alkaline phosphatase (IU/L) |  |  |  |  |  |  |
| ≤117 | 44 | Ref. |  |  | Ref. |  |
| >117 | 19 | 3.05 (1.60-5.82) | **<0.001** |  | 6.75 (2.89-15.78) | **<0.001** |
| Albumin (g/dL) |  |  |  |  |  |  |
| ≥3.6 | 57 | Ref. |  |  | Ref. |  |
| <3.6 | 6 | 1.47 (0.58-3.73) | 0.420 |  | 2.54 (0.75-8.60) | 0.135 |
| Lactate dehydrogenase (IU/L) |  |  |  |  |  |  |
| ≤240 | 21 | Ref. |  |  | Ref. |  |
| >240 | 3 | 1.86 (0.53-6.51) | 0.333 |  | 2.49 (0.30-21.09) | 0.402 |

ECOG: Eastern Cooperative Oncology Group; ARSi: androgen receptor signaling inhibitor; HR: hazard ratio; CI: confidence interval.


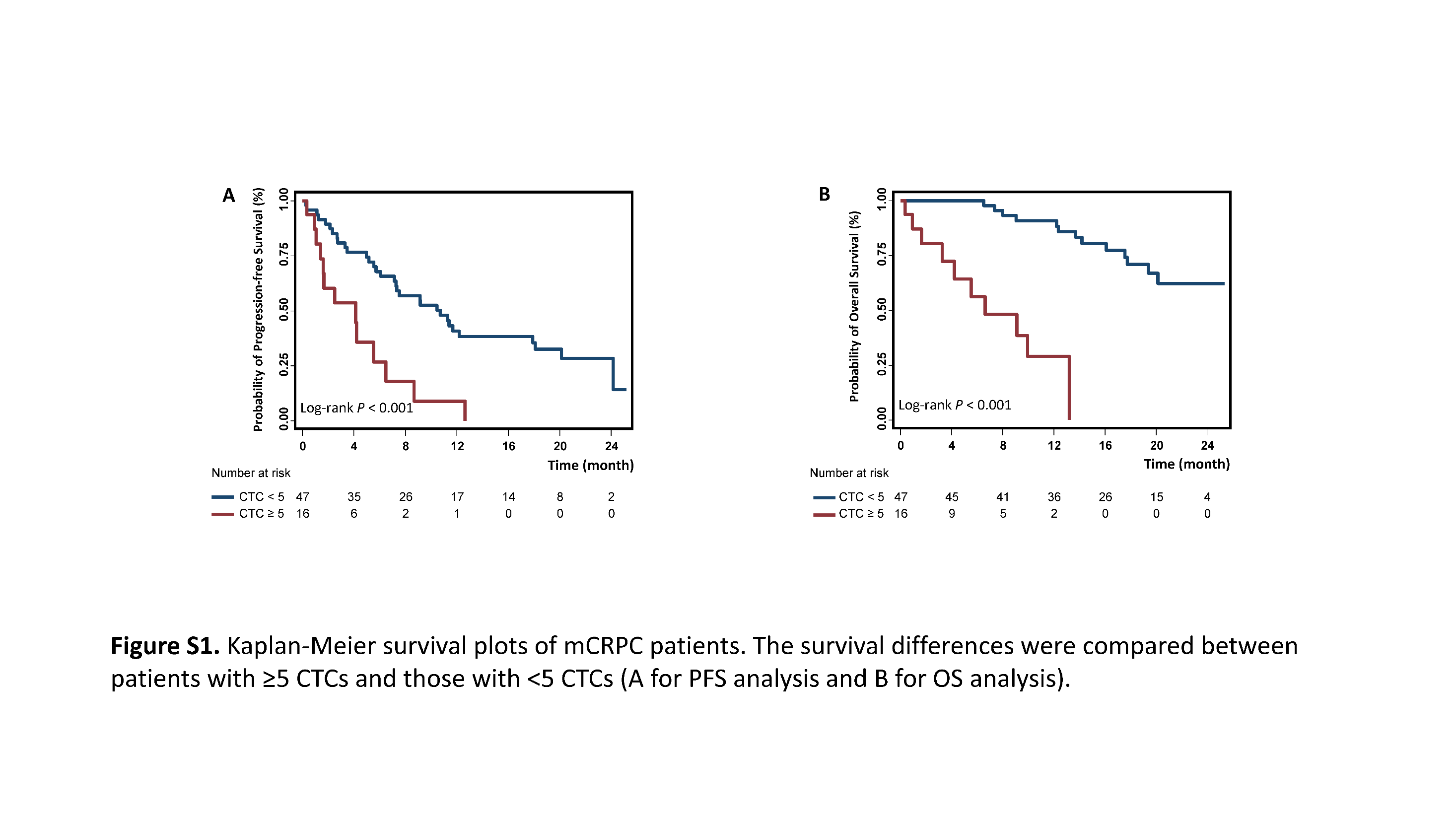

Supplement: Supplementary file 1 — Additional file 1: Table S1. Univariate analysis of associations with clinical outcomes. Figure S1. Kaplan-Meier survival plots of mCRPC patients. The survival differences were compared between patients with ≥5 CTCs and those with < 5 CTCs (A for PFS analysis and B for OS analysis). [file 12885_2021_8405_MOESM1_ESM.docx]
